# A Retrospective Review and Comprehensive Tumour Profiling of Advanced Non-Melanomatous Cutaneous Spindle Cell Neoplasms Treated with Immune-Checkpoint Inhibitors

**DOI:** 10.3390/cancers16081452

**Published:** 2024-04-10

**Authors:** Luke S. McLean, Annette M. Lim, Christopher Angel, Richard J. Young, Angela Pizzolla, Stuart Archer, Benjamin J. Solomon, Alesha A. Thai, Jeremy Lewin, Danny Rischin

**Affiliations:** 1 The Sir Peter MacCallum Department of Oncology, University of Melbourne, Melbourne, VIC 3052, Australia; lukes.mclean@petermac.org (L.S.M.);; 2 Department of Medical Oncology, Peter MacCallum Cancer Centre, Melbourne, VIC 3052, Australia; 3 Department of Pathology, Peter MacCallum Cancer Centre, Melbourne, VIC 3052, Australia; 4 Research Division, Peter MacCallum Cancer Centre, Melbourne, VIC 3052, Australia; 5 Monash Bioinformatics Platform, Melbourne, VIC 3168, Australia

**Keywords:** immune-checkpoint inhibitors, immunotherapy, cutaneous spindle cell neoplasm, biomarkers

## Abstract

**Simple Summary:**

Non-melanomatous cutaneous spindle cell neoplasms comprise a rare group of tumours, and in the advanced disease setting, a consensus on treatment approaches is lacking. These tumours are more common with age and have a propensity to develop in chronic ultraviolet (UV)-exposed sites. High response rates have been seen with immune-checkpoint inhibitors (ICI) in other UV-driven tumours such as cutaneous squamous cell carcinoma, yet these tumours have historically been excluded from the key clinical trials of immunotherapy. Patients with advanced non-melanomatous cutaneous spindle cell neoplasms have traditionally been offered chemotherapy for which response rates are variable and short-lived. Due to the impressive and durable response rates seen in our cohort of patients, clinicians should consider the use of ICIs in this heterogenous group of tumours. UV signatures and tumour mutational burden, if available, could serve as important biomarkers in the selection of patients for treatment with ICIs.

**Abstract:**

Non-melanomatous cutaneous spindle cell neoplasms are a rare group of malignancies that present a diagnostic challenge, and for which there is a lack of consensus on how to best manage patients with advanced disease and only limited reports of immune-checkpoint inhibitor (ICI) responses. In this study, we performed a single-center retrospective review of treatment outcomes for all advanced non-melanomatous cutaneous spindle cell neoplasms treated with ICIs. Blinded histopathology reviews occurred to confirm each diagnosis. Comprehensive tumour profiling included whole exome sequencing for tumour mutational burden (TMB) and ultraviolet(UV) signatures, and immunohistochemistry for immune-cell infiltration (CD4/CD3/CD8/CD103/CD20) and immune-checkpoint expression (PD-L1/LAG3/TIGIT). Seven patients were identified. The objective response rate was 86% (6/7) with five complete responses (CR). Responses were durable with two patients in CR > 30 months after ICI commencement. All patients had high TMB and UV signatures. One patient had PD-L1 100% (combined positive score) with abundant immune-cell infiltration and LAG3 expression. In advanced non-melanomatous cutaneous spindle cell neoplasms, excellent responses to ICIs with durable disease control were observed. ICIs are worthy of further exploration in these patients. UV signatures and high TMB could be used to help select patients for treatment.

## 1. Introduction

Cutaneous spindle cell neoplasms represent a diagnostic challenge for the pathologist, comprising a heterogenous group of tumours of mesenchymal and non-mesenchymal origins, overlapping morphological features and variable patterns of immunohistochemical staining [1]. This broad group of tumours can encompass spindle cell squamous cell carcinoma (SpSCC), pleomorphic dermal sarcoma (PDS), desmoplastic melanoma, angiosarcoma, and leiomyosarcoma. Poor biopsies and small excisions often add to the diagnostic difficulties faced by pathologists. However, the identification of the correct histopathology is important as it can carry prognostic significance. For example, local recurrences in patients with PDS can occur in up to 28% of cases within 2 years of resection with 20% then potentially developing metastatic diseases [2,3,4,5,6]. This is in contrast to a 5-year recurrence risk of 10.3% for local atypical fibroxanthoma [7].

In the advanced disease setting, immune-checkpoint inhibitors (ICI) have become the first-line systemic therapy of choice in the management of ultraviolet(UV)-induced tumours melanoma and cutaneous squamous cell carcinoma (CSCC), with a significant survival advantage, improvements in quality of life, and evidence of durable disease control [8,9,10,11,12]. Thus, increasing importance has been placed on ensuring biopsies of metastatic cutaneous spindle cell neoplasms are thoroughly interrogated to exclude melanoma or CSCC. For example, desmoplastic melanoma (a rare variant of malignant melanoma characterised by a spindle cell pattern with stromal fibrosis) is exquisitely sensitive to ICIs, whilst various sarcomas such as leiomyosarcoma are traditionally managed with chemotherapy [13]. There are also a small number of reports of SpSCC, a rare variant of CSCC, responding to ICIs [14,15]. However, despite the propensity for SpSCC to develop in UV-exposed sites, the presence of spindle cells, even in association with squamous cells, excluded these patients from the registrational cemiplimab clinical trials in CSCC [9].

Clearer definitions and a better understanding of the molecular and genomic landscape of cutaneous spindle cell neoplasms are also important to identify potential new treatment approaches. As an example, PDS tends to develop in UV-exposed sites and is often associated with a high tumour mutational burden (TMB), and it has been shown to harbour inflammatory/pro-immunogenic features such as tumour-infiltrating lymphocytes (TILs) [16,17,18,19]. These are features similar to other skin cancers such as melanoma and CSCC which are sensitive to ICIs and small reports have suggested patients with advanced PDS may have durable responses to ICIs [16,17,18,19]. With the exception of desmoplastic melanoma, where ICIs are standard of care, traditional treatment paradigms typically suggest cytotoxic chemotherapy for patients with advanced non-melanomatous cutaneous spindle cell neoplasms with variable efficacy, and for which is often precluded or poorly tolerated in the elderly cohorts predisposed to developing these malignancies.

In this study, we performed a retrospective review and comprehensive tumour profiling of all the advanced non-melanomatous cutaneous spindle cell neoplasms treated in our centre with ICIs. We aimed to demonstrate that advanced cutaneous spindle cell neoplasms arising from UV-exposed sites can be effectively treated with ICIs, highlight the diagnostic difficulties faced by pathologists in this heterogenous group of tumours and through comprehensive tumour profiling (TMB, UV signatures, immune cell infiltration, or immune-checkpoint expression), show that there is biological rationale for using immunotherapy. This is, to the best of our knowledge, the largest report on an advanced cohort of these tumours treated with ICIs to date.

## 2. Materials and Methods

### 2.1. Patients

This was a single-centre retrospective audit of patients treated between 5 May 2017, and 23 May 2022, with locally advanced (not amenable to curative radiotherapy or surgery post discussion in a multidisciplinary setting) or metastatic (nodal and/or distant) non-melanomatous cutaneous spindle cell neoplasms who received cemiplimab via an access scheme or who self-funded pembrolizumab. All patients were identified from a database of patients deemed ineligible for the Phase II multi-cohort registration study (NCT02760498) of cemiplimab in advanced CSCC. Clinicopathological data and formalin-fixed paraffin-embedded (FFPE) tumour blocks were retrospectively collected. Response rates were collected as per Response Evaluation Criteria in Solid Tumors (RECIST1.1) [20] if followed by computed tomography (CT) or Positron Emission Tomography Response Criteria (PERCIST1.0) [21] if followed by fluorodeoxyglucose positron emission tomography (FDG-PET/CT). Objective response rate (ORR) was defined as the number of complete responses and partial responses as per these criteria divided by the overall number of response assessments.

This study was reviewed and approved by the Human Research Ethics Committee (HREC) (HREC/76580/PMCC and HREC/73275/PMCC) of the Peter MacCallum Cancer Centre, Melbourne, Australia. This project was conducted in accordance with the Declaration of Helsinki.

### 2.2. Histopathology and Immunohistochemistry

All non-melanomatous cutaneous spindle cell neoplasms included in this study were initially identified by a pathologist on routine diagnostic hematoxylin-eosin (H&E) review. The H&E-stained slides for these cases were then reviewed by a pathologist with expertise in cutaneous malignancies (CA). In each case, the pathologist was blinded to historical histopathology reports. All cases were reviewed to exclude melanomatous spindle cell malignancies. A sub-diagnosis for each spindle cell tumour was obtained where possible on the basis of morphology and immunohistochemical findings.

A broad diagnostic immunohistochemistry (IHC) panel was utilised encompassing epithelial (AE1/3, HMWCK), squamous (CK56, p40, p63), melanoma (SOX-10, melan-A), muscle differentiation (SMA, desmin), endothelial (CD31, ERG), and mesenchymal (PDGFRA/B) markers. CD34 was included to assist in the identification of vascular and other soft tissue malignancies. CD10 was included as strong diffuse staining is very common in PDS, and its absence usually supports another diagnosis [22]. Additional exploratory IHC aimed at characterising the immune tumour microenvironment (TME) was performed utilising T cell markers (CD3, CD4, CD8, CD103), a B cell marker (CD20), and immune checkpoints (PD-L1, LAG3, TIGIT) [23,24]. Programmed death-ligand 1 (PD-L1) (22C3) staining was scored as a combined positive score (CPS) [25]. Details on the antibodies used, the protocol for staining and IHC scoring, are provided in Supplementary S1.

### 2.3. DNA and RNA Isolation

For both DNA and RNA extractions, 4 × 10 μm sections were prepared from FFPE blocks. The area of tumour was marked by a pathologist using H&E-stained slides with marked normal and tumour regions and macro-dissected. Extractions were only performed if tumour cellularity was >20%.

RNA was extracted utilising RNeasy FFPE Kits (Qiagen, Hilden, Germany) and samples were treated with DNase digestion. Tumour DNA was extracted using a QIAamp DNA FFPE Kit (Qiagen). For germline DNA patients either had FFPE blocks reviewed by a pathologist for regions of normal tissue that were subsequently marked and macro-dissected for DNA extraction, or it was extracted from freshly collected blood samples (peripheral blood mononuclear cells (PBMC)) using an All-Prep DNA/RNA Microkit (Qiagen). DNA was quantified using Qubit dsDNA BR Assay and RNA was quantified using the Agilent TapeStation 4150.

### 2.4. RNA Sequencing

First, 200 ng of purified RNA were depleted for ribosomal RNA via *NEBNext rRNA Depletion Kit (Human/Mouse/Rat)* before library preparation via *NEBNext Ultra II Directional RNA Library Prep Kit for Illumina* as per manufacturer protocols. Final libraries were sequenced on *NextSeq2000* in paired-end 100 bp configuration to a mean of 100-million paired reads per sample. Samples were demultiplexed and converted to fastq using *bcl2fastq2* (Version 2.20). For the SingScore analysis, RNA-sequencing reads were first aligned to the human transcriptome (GRCh38; transcript annotation from Ensembl v109), and expression values were estimated as transcripts per million (TPM) using Salmon 1.9.0 [26], as implemented in the nf–core–rnaseq pipeline v-3.10.1 [27]. These values were aggregated to per-gene TPM, and any genes that did not align more than 10 read pairs in at least one sample were excluded from further analysis. SingScore rank scores for the 18-gene interferon–gamma gene signature [28] were then calculated from the per-gene TPM values using SingScore v.1.16.0 [29]. The list of genes analysed included: CD3D, IDO1, CIITA, CD3E, CCL5, GZMK, CD2, HLA-DRA, CXCL13, IL2RG, NKG7, HLA-E, CXCR6, LAG3, TAGAP, CXCL10, STAT1, and GZMB.

### 2.5. Whole-Exome Sequencing (WES)

Up to 300 ng of purified DNA were fragmented to ~200 bp via *Covaris LE220* using previously optimised shearing conditions for FFPE inputs. Library fragments were prepared via *KAPA HyperPrep* using *Agilent SureSelect XT HS* adaptors and pre-capture primers. Exome capture was performed as per *Agilent SureSelect XT HS* manufacturer protocols using the *Agilent Clinical Research Exome V2* bait panel. Final libraries were sequenced on *NextSeq500* in paired-end 75 bp configuration to a mean coverage of 80× for PBMC germline samples and 150× for FFPE tumour samples. Samples were demultiplexed and converted to fastq using *bcl2fastq2* (version 2.20) and analysed using the nf–core Sarek-v3.1.2 pipeline [30] against the GRCh38 human reference genome (Ensembl). Briefly, initial alignment was performed using BWA-MEM v0.7.17 [31], and base-score recalibration was conducted using the GATK4 best-practices workflow with GATK4.4.3 [32]. Variant calling was also conducted using the Sarek v3.1.2 pipeline using GATK-Mutect2, and snpEff v5.1 [33] was used to characterise the variants.

### 2.6. Tumour Mutational Burden (TMB) and Signature Analysis

Calculation of TMB occurred using pyTMB (v1.3, Curie Institute, Paris, France) on the variant call files with an effective genome size of 33.28 megabases. This was done using the standard pyTMB parameters on the variant files after calling variant effects using snpEff. Single-substitution mutation signatures in triplet context were counted and assigned to COSMIC signatures using the mutSignatures R package (R Version 4.2.1) [34]. Substitutions were used to calculate the contributions of known mutation signatures (SBS1-30, including sub-signatures SBS7a and SBS7b) from the COSMIC v3.3.1 database [35] towards the somatic mutation spectrum observed in each tumour sample. For further detail please see Supplementary S1.

## 3. Results

### 3.1. Patient Characteristics

Histopathology reports for 80 patients were reviewed. We identified seven patients with advanced non-melanomatous cutaneous spindle cell neoplasms treated with ICIs. The median age was 71.9 years (range: 66–79), and all primaries originated in UV-exposed sites of the head and neck region (three scalp, two cheek, one temple, one preauricular). Three patients had distant metastatic disease, four patients were immunocompromised, and ICIs represented the first-line systemic therapy agent for all patients. Patient characteristics are summarised in Table 1.

### 3.2. Response Assessments

**Figure 1:**
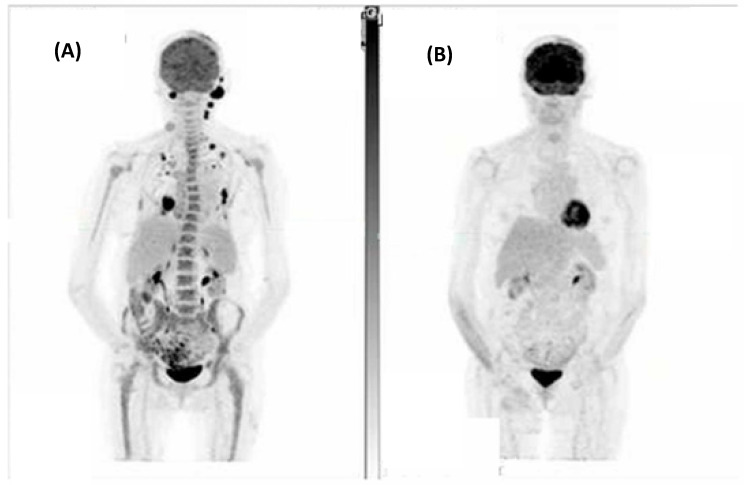
A patient with metastatic spindle-cell cutaneous squamous cell carcinoma involving the scalp, left occipital and neck nodes, right chest wall, and left adrenal with bilateral pulmonary metastases. (**A**) Fluorodeoxyglucose positron emission tomography (FDG-PET) at baseline and (**B**) Post 7 cycles of cemiplimab demonstrating a complete metabolic response to treatment.

The combined ORR for this cohort was 86% (6/7), with five (71%) patients achieving either a complete metabolic response (CMR) on FDG-PET/CT or complete response (CR) on CT as their best response. Figure 1 demonstrates a CMR that was achieved for one patient. Of the two patients who did not achieve a CMR or CR, one achieved a partial metabolic response and the other stable metabolic disease (SMD) on FDG-PET/CT.

**Figure 2:**
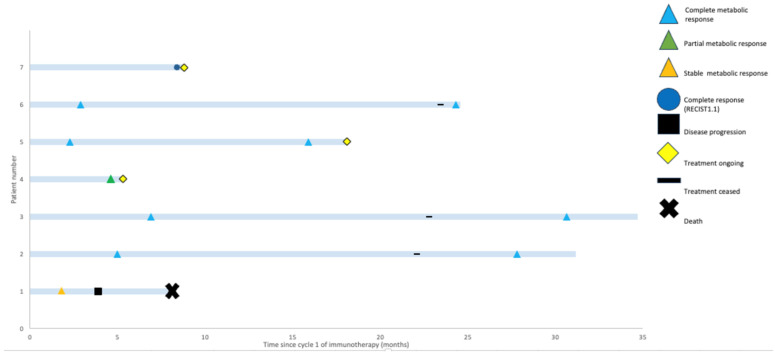
Swimmers’ plot highlighting potential for durable responses to immunotherapy. Best-response assessment, most recent-response assessment, treatment status, and survival are shown for each patient.

With a median follow up of 18.1 months (range: 5.3–34.7 months), only the patient with SMD progressed (Figure 2). This patient had an SpSCC with intracranial extension and, on initial review, had significant ataxia requiring a mobility aid. After two cycles of cemiplimab, they experienced improvement in their symptoms and were able to walk independently with SMD on imaging. However, after eight cycles of immunotherapy, they experienced progression in both the intracranial and extracranial components of their disease and declined functionally, requiring admission to a palliative care unit.

### 3.3. Diagnostic Immunohistochemistry

To better understand the histopathological features of this group of tumours and to retrospectively clarify histologic subtypes, we performed comprehensive IHC, which is summarised in Table 2. Three cases were diagnosed as SpSCC. These tumours demonstrated both spindle cell and squamous cell morphologies on H&E with the expression of squamous and epithelial markers. Two further cases were diagnosed as PDS demonstrating no specific immunohistochemical reactions other than strong diffuse staining for CD10 but characteristic morphological features of a pleomorphic predominantly spindle cell lesion. For two patients, the histologic sub-type was less clear.

Patient 4 was referred for the consideration of systemic therapy for what was originally diagnosed as a recurrent PDS in the neck. Further radiotherapy was not possible since the patient received post-operative radiotherapy after the removal of the original primary tumour, and surgery was expected to result in the loss of function to the arm. Our IHC panel identified that this tumour was also p63 positive, which whilst not specific, does raise the possibility of a de-differentiated SpSCC.

Patient 5 presented with 2 months of right facial pain and swelling and was found to have a lesion over the parotid with involvement of the pterygoid muscles, extension as far as the pterygopalatine fossa, and multiple involved neck nodes. IHC was non-specific but did reveal diffuse positive staining for desmin. Whilst a definitive sub-diagnosis could not be reached and was in particular limited by the small amount of diagnostic tissue available, leoimyosarcoma was raised as a possible diagnosis for this lesion.

### 3.4. Genomic Characterisation, TMB and Analysis for UV-Associated Mutational Signatures

**Figure 3:**
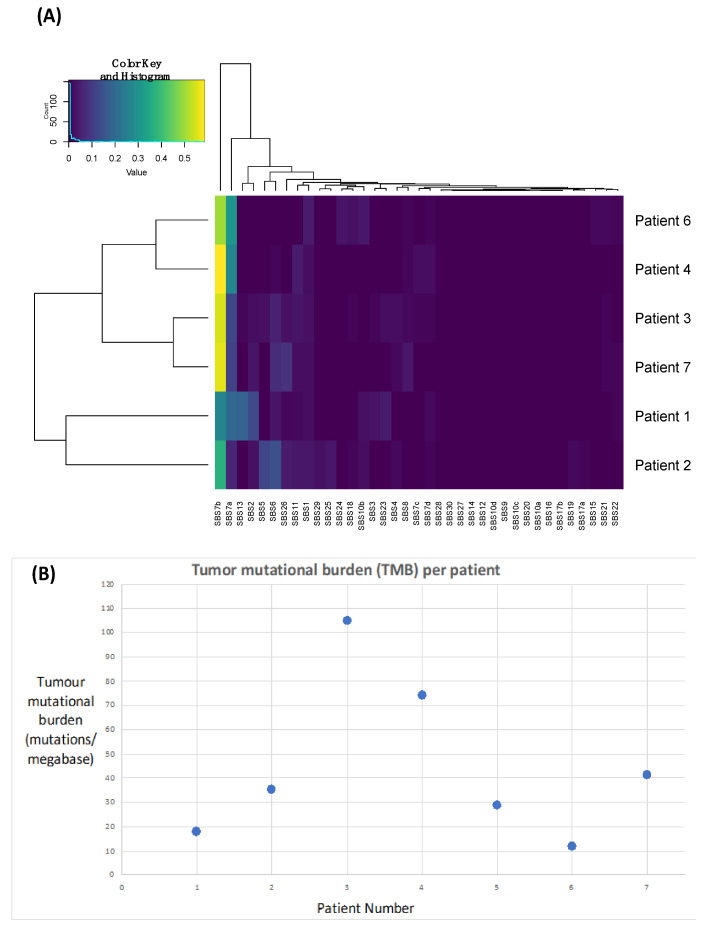
Plots highlighting the presence of Ultraviolet Signatures 7a and 7b (**A**) and an elevated tumour mutational burden (**B**) across all patients.

In order to demonstrate that these tumours are likely driven by chronic UV exposure, we performed signature analyses. UV-induced signatures 7a and 7b were present in all samples (Figure 3A, Table 3), supporting this hypothesis. Signature 13 and 2 were also identified in Patient 1, which has been linked to APOEBEC deficiency and may also be associated with improved responses to immunotherapy [36].

We next calculated TMB as a surrogate of the neoantigen load, which we anticipated would be high due to excessive UV light-driving DNA damage and a hypermutated phenotype. We identified a median TMB of 35.3 nonsynonymous variants per megabase (range: 12.02–104.87) (Figure 3B, Table 3).

**Figure 4:**
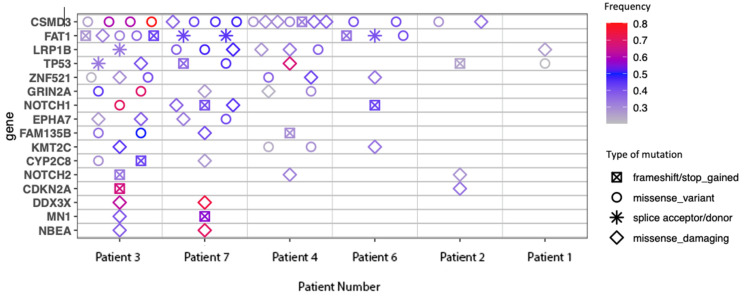
Commonly mutated genes.

Commonly mutated genes are shown in Figure 4. This list features mutations commonly seen in other cutaneous malignancies, in particular, mutations in CDKN2A and TP53, which are also frequently seen with chronic UV exposure [37]. CSMD-3 and FAT1 mutations were also identified, which have been shown to correlate with high TMB [38,39].

### 3.5. Exploratory Immune Cell and Immune Checkpoint Immunohistochemistry

We next investigated whether these tumours had an infiltration of T cells (CD103, CD4, CD3, and CD8) and B cells (CD20) or expressed immune checkpoints (PD-L1, TIGIT, and LAG3) (Table 3) as these features are known to positively correlate with a response to immunotherapy [40,41]. Patient 2 demonstrated a moderate infiltration of CD3+, CD4+, and CD8+ cells. In all cases, CD20 was negative and CD103 was lowly expressed, suggesting no B cells and low tissue-related memory T cells, respectively. The IHC for immune cell markers otherwise demonstrated minimal expression across the other five cases. In terms of checkpoint expression, Patient 2 demonstrated a high PD-L1 CPS score of 100 and significant LAG3 expression. TIGIT was not expressed in any samples.

### 3.6. SingScore for Interferon–Gamma Signatures

Interferon–gamma signatures have been found to correlate with immunotherapy responses [28], and we hypothesised that they may be elevated in these tumours. SingScore values for an interferon–gamma signature in this cohort were heterogenous. Patient 2 demonstrated a higher score with a low level of dispersion, indicating that most of the tested genes had an expression that was higher than the median gene expression across all genes. This patient also demonstrated a deep and durable CMR to treatment with an ongoing response > 30 months post-immunotherapy commencement. However, for the other cases, dispersion was higher, indicating that for these sample expressions in the gene set varied, but not in a concordant way, and thus was not supportive of interferon–gamma pathway activation. (Supplementary Figure S1).

## 4. Discussion

This study presents encouraging clinical activity for the use of ICIs in the management of advanced non-melanomatous cutaneous spindle cell neoplasms with 86% (6/7) patients achieving responses that appear durable to treatment. Analyses demonstrated that all cases had evidence of UV-mutational signatures and elevations in TMB, which are similar features to other UV-associated skin cancers where ICIs are the established first-line treatment for advanced diseases. However, interestingly, further analysis with IHC and RNA sequencing did not uniformly demonstrate an inflammatory phenotype.

Non-melanomatous cutaneous spindle cell malignancies are more frequent with increasing age and in immunocompromised patients, which was also reflected in the demographics of our cohort. There are often concerns about the tolerability of chemotherapy in elderly and immunocompromised patients and, thus, the exploration of the efficacy of ICIs in these patients is desirable. In our cohort, four patients were immunocompromised, a group where there have been historical concerns about reduced response rates to ICIs [42]. However, despite this, durable responses were seen in three out of four (75%) of these patients. This cohort was not selected based on treatment response and rather represents all treated cases in our centre. Thus, the fact that all patients derived benefit and 6/7 (86%) patients demonstrated a response to ICIs is impressive. These patients were all referred to our unit with an advanced incurable malignancy and limited treatment options on the basis of their histology. It is, therefore, clinically significant that none of those that responded have progressed to date and that two remain off ICIs and in CR beyond 30 months (Figure 2).

Despite advanced non-melanomatous cutaneous spindle cell malignancies comprising a heterogenous histological group, high response rates to ICIs were seen in our cohort. In the literature, there are some reports of responses to ICIs in advanced PDS and in undifferentiated pleomorphic sarcoma harbouring UV-mutational signatures and elevations in TMB [6,18,43]. There are also two case reports on advanced SpSCCs, a rare variant of CSCC, responding to ICIs [14,15]. Whilst a recognised variant of CSCC, SpSCCs were unfortunately excluded from the key cemiplimab trials in advanced CSCC [9]. Durable responses are well documented in other immunotherapy-responsive cutaneous malignancies such as metastatic melanoma and CSCC [10,44,45]. However, reports are lacking in advanced non-melanomatous cutaneous spindle cell tumours.

The diagnosis of these tumours remains a challenge. Whilst morphologically the identification of spindle cells may be straightforward, further categorisation of histologic subtypes can prove difficult. Guidelines have been developed to assist pathologists and acknowledge that it is not always possible to classify these lesions with absolute certainty and, thus, a descriptive diagnosis representative of all available information is often required [1,46]. Despite the use of a broad IHC panel and expert pathologist review, only 5/7 cases had a final histologic subtype that was confidently agreed upon. PDGFR-B has recently been proposed as a marker of mesenchymal differentiation to help distinguish PDS from CSCC [16]. We, therefore, included this in our IHC panel. However, for our two advanced PDS tumours, PDGFR-B was negative. Clarifying the histologic subtype is of utmost importance when the histotype drives treatment choices. For example, desmoplastic melanoma is a melanomatous spindle cell neoplasm that is exquisitely sensitive to ICIs, and for which ICIs are standard of care. Fortunately, this tumour is often easy to distinguish from its non-melanomatous counterparts on the basis of melanocytic marker expression (i.e., HMB-45, Melan-A) and molecular profiling [1]. All seven of the tumours in this report arose from UV-exposed sites, had UV-mutational signatures, and an elevated TMB. Thus, these features, rather than histology alone, could perhaps be used to help select patients for ICIs. We believe this is an approach that will be worthy of consideration in the design of future prospective studies.

Some traditional features of immunotherapy responsiveness have included the presence of an inflammatory cell infiltrate, expression of immune checkpoints, and activation of interferon–gamma pathways [28,47,48,49]. Whilst these pathways have been validated in other immunotherapy-responsive tumours, they have not been looked at in this particular group. There are also some data in PDS suggesting these tumours may have an inflamed TME^15^. We thus hypothesised that our samples would have high levels of inflammatory cells, expression of checkpoints, and an interferon–gamma signature. However, this was not the case. Results were heterogenous and, overall, largely negative for these parameters. There was one patient who had a very high PD-L1 expression (CPS of 100), elevated LAG3 expression, and evidence of intratumoural T cell infiltration. This was in stark contrast to the rest of the cohort, which suggests that multiple other factors can exist that contribute to ICI responses. TMB has also been shown to be a predictive marker of ICI responses in other cancers [50] and was uniformly elevated in all of our cases. The mutational profile in our cases was also similar to what has been reported in other cutaneous malignancies, including mutations associated with chronic UV exposure (such as TP53 and CDKN2A) and UV-light exposure-associated signatures [37]. These features are also common to UV-induced tumours malignant melanoma and advanced CSCC.

This study was limited by its retrospective nature and small sample size. However, this must be interpreted in the context of this being a rare group of tumours, for which literature is limited. These findings are also limited to spindle cell cutaneous malignancies arising in the UV-exposed head and neck region. There was also no central assessment of disease response nor uniformity in choice of imaging. Additionally, because of the impressive response rates to ICIs in these patients, there was no cohort of immunotherapy-resistant tumours to contrast biological features of immunotherapy response and resistance.

## 5. Conclusions

In this study, we demonstrated high upfront response rates to ICIs and a potential for durable disease control in patients with advanced non-melanomatous cutaneous spindle cell malignancies originating from sun-exposed sites. This study suggests ICIs are worthy of further exploration in the management of patients who develop this malignancy. All these tumours harboured UV-exposure-associated mutational signatures and elevated TMB, which could be used to help select patients in a prospective study. Further research is required to clarify biological predictors of immunotherapy response in these patients.

## Figures and Tables

**Table 1:** Patient characteristics.

Case	Age (Years), Sex	Site of Disease	Stage	Treatment Received	Best Response on Imaging *	Immunocompromised	Prior Systemic Therapy	Progressed?	Pathology Review Final Diagnosis
1	73, male	Scalp	Locally advanced without nodal involvement	Cemiplimab	Stable metabolic disease	Chronic lymphocytic leukaemia	No	Yes, after 8 cycles	Spindle cell cutaneous squamous cell carcinoma
2	79, female	Scalp	Metastatic	Cemiplimab	Complete metabolic response	No	No	No	Spindle cell cutaneous squamous cell carcinoma
3	66, male	Scalp	Metastatic	Cemiplimab	Complete metabolic response	Multiple myeloma	No	No	Pleomorphic dermal sarcoma
4	66, female	Cheek	Locally advanced with nodal involvement	Cemiplimab	Partial metabolic response	Follicular lymphoma	No	No	Cutaneous spindle cell malignancy—subclassification unclear
5	75, male	Cheek	Locally advanced with nodal involvement	Cemiplimab	Complete metabolic response	No	No	No	Cutaneous spindle cell malignancy—subclassification unclear
6	70, male	Temple	Locally advanced with nodal involvement	Cemiplimab	Complete metabolic response	No	No	No	Spindle cell cutaneous squamous cell carcinoma
7	74, male	Preauricular	Metastatic	Pembrolizumab	Complete response	Chronic lymphocytic leukaemia	No	No	Pleomorphic dermal sarcoma

* Patients 1–6 were followed with fluorodeoxyglucose positron emission tomography (FDG-PET) and assessed as per Positron Emission Tomography Response Criteria (PERCIST1.0). Patient 7 was followed with computed-tomography (CT) and assessed as per Response Evaluation Criteria in Solid Tumors (RECIST1.1).

**Table 2:** Diagnostic immunohistochemistry * panel for each patient.

	Epithelial and Squamous Differentiation					Melanomatous Differentiation		Muscle Differentiation		Endothelial Differentiation		Other		Mesenchymal Differentiation	
Patient No. + Histology	AE1/3	HMWCK	CK5/6	p40	p63	SOX10	Melan-A	SMA	Desmin	CD31	ERG	CD34	CD10	PDGFRA	PDGFRB
1 *SpSCC*	80%+	80%+	80%+	80%+	80%+	-	-	80%+	-	-	-	-	80%+	-	-
2 *SpSCC*	80%+	80%+	80%+	60%	80%+	-	-	-	-	60%	-	-	80%+	-	-
3 *PDS*	-	-	-	-	-	-	-	-	-	-	-	-	80%+	-	-
4 *Unclear SpN*	-	-	-	-	80%+	-	-	10%	-	-	-	-	80%+	-	-
5 *Unclear SpN*	-	-	-	-	-	-	-	-	80%+	-	-	-	-	-	80%+
6 *SpSCC*	-	30%	30%	50%	70%	-	-	-	-	-	-	-	-	-	-
7 *PDS*	-	-	-	-	10%	-	-	-	-	-	-	-	80%+	-	-

* Immunohistochemistry scores are a percentage of positively stained cells. SpSCC—Spindle cell cutaneous squamous cell carcinoma, PDS—Pleomorphic dermal sarcoma, Unclear SpN—Cutaneous spindle cell malignancy of unclear subclassification.

**Table 3:** Immunohistochemistry ^, tumour mutational burden, and ultraviolet signatures.

	T-Cell Markers				B-Cell Markers	Immune-Checkpoints			Tumor Mutational Burden (mt/mb)	Ultraviolet-Mutational Signature (7a/7b)	Best Response
Patient											Assessment ^#^
	CD103	CD4	CD3	CD8	CD20	PD-L1	TIGIT	LAG3			
1	1	20	1	1	0	7	0	0	17.94	Yes	Stable metabolic disease
2	1	20	30	10	0	100	0	10	35.34	Yes	Complete metabolic response
3	1	10	10	5	0	5	0	0	104.87	Yes	Complete metabolic response
4	1	0	1	0	0	0	0	0	74.04	Yes	Partial metabolic response
5 *									29	Yes	Complete metabolic response
6	1	0	0	5	0	0	0	4	12.02	Yes	Complete metabolic response
7	1	0	5	1	0	0	0	0	41.17	Yes	Complete response

^ IHC scores are a percentage of positively stained cells, except for PD-L, which is combined positive score (CPS). * Insufficient tissue for immunohistochemistry on Patient 5. # As per PERCIST1.0 on FDG-PET/CT or RECIST1.1 on CT.

## Data Availability

The datasets generated and analysed in this study are available from the corresponding author upon reasonable request.

## Supplementary Materials

The following supporting information can be downloaded at: https://www.mdpi.com/article/10.3390/cancers16081452/s1, Supplementary S1. Supplementary Methodology; Figure S1. SingScores; Table S1. Immunohistochemistry.
