# Supplementary material for: A Retrospective Review and Comprehensive Tumour Profiling of Advanced Non-Melanomatous Cutaneous Spindle Cell Neoplasms Treated with Immune-Checkpoint Inhibitors
Source: Cancers (Basel). 2024 Apr 10;16(8):1452. doi: 10.3390/cancers16081452 (PMC11048307; doi:10.3390/cancers16081452)
Supplement: Supplementary file 1 [file cancers-16-01452-s001.zip › cancers-2865245-supplementary.pdf]

Supplementary Materials: A Retrospective Review and Comprehensive Tumour Profiling of Advanced Non-Melanomatous Cutaneous Spindle Cell Neoplasms Treated with Immune-Checkpoint Inhibitors

Luke S. McLean, Annette M. Lim, Christopher Angel, Richard J. Young, Angela Pizzolla, Stuart Archer, Benjamin J. Solomon, Alesha A. Thai, Jeremy Lewin and Danny Rischin

Supplementary S1 Supplementary Methodology

Diagnostic Immunohistochemistry

Immunohistochemistry (IHC) for pathological/diagnostic markers AE1/3, HMWCK, CK56, p40, p63, SOX-10, melan-A, SMA, desmin, CD31, ERG, CD10 , CD34, and PDGFRA was performed on a Ventana Ultra Benchmark platform (Roche Diagnostics) on 4μm formalin-fixed paraffin-embedded (FFPE) tumour sections utilizing the following method: slides were de-waxed in EZ Prep Concentrate (10X) (Roche; cat# 05279771001) prior to antigen retrieval in Cell Conditioning solution 1 (Roche; cat# 905424569001) after which slides were incubated in pre-primary peroxidase inhibition solution, followed by primary antibody incubation at 36^{0}C using applicable working dilutions and incubation times optimised for each individual antibody. Slides were processed using complementary OptiView DAB IHC Detection Kit (Roche; cat# 06396500001) after which slides were washed in Ezy Prep (Roche; cat# 05279771001) and loaded onto a Tissue-Tek Prisma Autostainer (Leica Biosystems) for counterstaining using a dehydrate, clear and mount protocol.

Immunohistochemistry for immune cells and checkpoints

Immunohistochemistry for immune cells and checkpoints was performed on a Dako autostainer (Agilent) on 4μm FFPE tumour sections utilizing the following method (except programmed death-ligand 1 (PD-L1) 22C3): sections were de-waxed prior to antigen retrieval using DAKO high pH target retrieval solution (Agilent; cat# K8006) in a pressure cooker. Slides were then placed onto the Dako autostainer for the following incubations: 3% H_2O_2 for 10 minutes; primary antibody for 60 minutes (see Table S1 for details); secondary detection with Envision+ HRP rabbit (Agilent; cat# K400311) or mouse (Agilent; cat# K4001) for 60 minutes; DAB (Agilent; cat# K346811) for colour reaction for 10 minutes. All slides were rinsed with TBST buffer between the above incubations. Slides were then removed from the autostainer, counterstained with hematoxylin and cover-slipped.

For PD-L1 (22C3) IHC was performed using the PD-L1 IHC 22C3 PHARMDX kit (Agilent), following the method provided in the kit.

Immunohistochemistry scoring

For each of CD3, CD4, CD8, CD103, CD20, TIGIT and LAG3 the abundance of positively stained immune cells was determined by semi-quantitatively scoring as a proportion of the total cells (including tumor cells, immune cells, others) within tumor areas as 0%, 1%, 5%, 10%, 20%, 30% - 100% (to the nearest 10% above 10%)[1,2]. MHC I and MHC II were scored as positive or negative within tumor cells. The remaining diagnostic IHC was reported as the percentage of positively stained tumor cells to the nearest 10%. PD-L1 (22C3) staining was scored according to the instructions in the assay kit for combined positive score (CPS), which is the number of positively stained cells (tumor cells, lymphocytes, macrophages) divided by the total number of viable tumor cells, multiplied by 100[3].

The block for Patient 5 was exhausted for the purposes of diagnostic IHC and thus IHC for immune cells and checkpoints was unable to be performed.

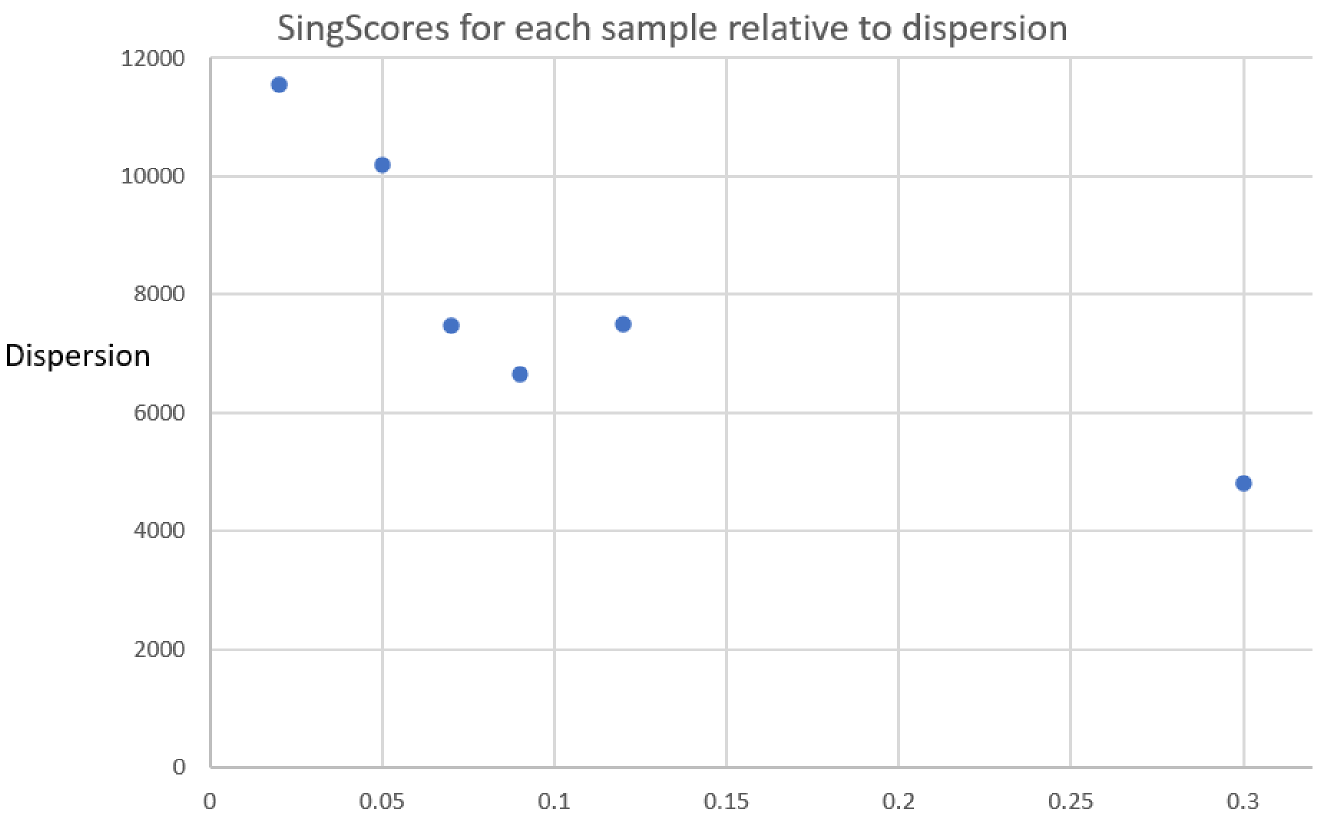

Figure S1. SingScores.

Table S1. Immunohistochemistry.

Antibody	Type	Antibody dilution	Antibody supplier (cat #)
XXXX		*i.e 1:500*	*i.e Leica Biosystems (#CD8-4B11-CE, SANTA BARBARA, CA, USA)*
CD3	Rabbit monoclonal	1:200	Abcam (ab16669)
CD4	Mouse monoclonal	1:100	Invitrogen (14-2444-82)
CD8	Mouse monoclonal	1:500	Leica (NCL-L-CD8-4B11)
CD103	Rabbit polyclonal	1:1500	Abcam (ab129202)
CD20	Mouse monoclonal	1:750	Agilent (M0755)
PDL1 (22C3)	Mouse monoclonal	Pre-dilute	Agilent (SK00621-205)
TIGIT	Rabbit monoclonal	1:400	Cell Signalling (99567T)
LAG3	Rabbit monoclonal	1:1500	Abcam (ab180187)
MHC I	Mouse monoclonal	1:10,000	Abcam (ab70328)
MHC II	Mouse monoclonal	1:400	Agilent (M0775)
AE1/3	Mouse monoclonal	1:200	LEICA (NCL-L-AE1/AE3)
HMWCK	Mouse monoclonal	1:200	Abacus (Cell Marque) CM334M85
CK56	Mouse monoclonal	RTU	VENTANA (6478441001)
P40	Mouse monoclonal	1:100	Biocare (METAGENE) ACI13121B
P63	Mouse monoclonal	1:40	Agilent (M731701)
Sox10	Rabbit monoclonal	RTU	VENTANA (7560389001)
Melan-A	Mouse monoclonal	1:50	Agilent (NCL-L-MELANA)
SMA	Mouse monoclonal	RTU	VENTANA (05268303001)
Desmin	Mouse monoclonal	1:200	Agilent (M076001-2)
CD34	Mouse monoclonal	1:50	Agilent (M716501-2)
CD31	Mouse monoclonal	1:100	Agilent (M0823)
ERG	Rabbit monoclonal	RTU	VENTANA (6478450001)
CD10	Mouse monoclonal	1:50	LEICA (CD10-270-L-CE)
PDGFRA	Mouse monoclonal	1:100	Santa Cruz (SC-398206)

PDGFR-B	Rabbit monoclonal	1:50	BioSB (BSB 2352)

Calculation of tumour mutational burden (TMB) and signature analyses for Patient 5

The block for Patient 5 was exhausted during diagnostic IHC. However prior to block exhaustion sufficient DNA had been isolated from tumor and been sent for whole genome sequencing (WGS). This sample failed quality control with poor sequence coverage (at 16x, below target of 100x). Given that low effective DNA coverage confounds sensitivity more than accuracy orthogonal evidence was sought from other assays to support findings for TMB and signature analyses.

Matching whole transcriptome sequencing (WTS) was scanned for robust evidence of the somatic variants detected in the failed WGS sample (pile-up analysis was used to identify somatic mutations with >5 RNA reads supporting the variant). Only these verified mutations were then considered for TMB and mutational signature detection. TruSight Oncology 500 next-generation sequencing assay (TSO-500;Illumina, San Diego, CA, USA) was also available on circulating tumor DNA from a freshly collected blood sample. Reportable WGS mutations were verified in the circulating tumor TSO500 data. Variants were annotated using the TSO-500 pipeline for synonymous variants and Cancer Genome Interpreter.

It was concluded that TMB was high (> 10 mutations (mut)/megabase (mb)):

- TMB called from WGS alone was 29 mut/mb
- TMB called from WTS verified variants was 12 mut/mb
- TMB called from ctTSO500 was 30 mut/mb

The dominant mutational signature called from WTS verified variants in a UV-signature (2781 mutations assigned; 56%).

References

1. Solomon B, Young RJ, Bressel M, et al. Identification of an excellent prognosis subset of human papillomavirus-associated oropharyngeal cancer patients by quantification of intratumoral CD103+ immune cell abundance. Ann Oncol. 2019;30(10):1638-46.
2. Solomon B, Young RJ, Bressel M, et al. Prognostic Significance of PD-L1(+) and CD8(+) Immune Cells in HPV(+) Oropharyngeal Squamous Cell Carcinoma. Cancer Immunol Res. 2018;6(3):295-304.
3. Agilent Technologies, PD-L1 IHC 22C3 pharmDx Interpretation Manual – Head and Neck Squamous Cell Carcinoma (HNSCC), 2019.
